# Supplementary material for: Nutritional Interventions to Improve Asthma-Related Outcomes through Immunomodulation: A Systematic Review
Source: Nutrients. 2020 Dec 16;12(12):3839. doi: 10.3390/nu12123839 (PMC7765612; doi:10.3390/nu12123839)
Supplement: Supplementary file 1 [file nutrients-12-03839-s001.pdf]

Table S1. Calculation of the Jadad score.

Cluster	First author (year)	Intervention	Q1	Q2	Q3	Q4	Q5	Total score
Herbs, herbal mixtures and extracts	Barlianto (2017)*<br>Barlianto (2018)*	*Nigella Sativa* oil	1	0	0	0	1	2
	Hosseini (2018)**<br>Zilaee (2019)**	Saffron	1	1	1	1	1	5
	Khayyal (2003)	Aqueous extract of propolis	1	0	0	0	1	2
	Koshak (2017)	*Nigella Sativa* oil	1	1	1	1	1	5
	Salem (2017)	*Nigella Sativa*	1	1	0	0	1	3
	Yugandhar (2017)	Extract of *B. serrata* gum resin and *A. marmelos* fruit	1	0	1	1	1	4
Supplements	Ghaffari (2014)	Vitamin E	1	0	1	0	1	3
	Pearson (2004)	Vitamin E	1	1	1	1	1	5
	Smith (2015)	Soy isoflavone	1	1	1	1	1	5
	Wood (2008)	Tomato extract and tomato juice	1	1	0	0	1	3
Weight loss	Dias-Junior (2014)	Low calorie intake, use of sibutramine and use of orlistat	1	1	0	0	1	3
	Jensen (2013)	Energy reduction and counselling sessions	1	1	0	0	1	3
	Toennesen (2018)	High protein + low glycemic index diet and combination of diet and exercise	1	1	0	0	1	3
Vitamin D3	Bar Yoseph (2015)	Vitamin D3	1	0	1	1	1	4
	Castro (2014)	Vitamin D3	1	1	1	1	1	5
	de Groot (2015)	Vitamin D3 (Cholecalciferol)	1	0	1	1	1	4
	Kerley (2016)	Vitamin D3	1	0	1	0	1	3
	Martineau (2015)	Vitamin D3 (Vigantol oil)	1	1	1	1	1	

Omega-3 LCPUFA	Emelyanov (2002)	Lipid extract of the New Zealand green-lipped mussel	1	1	1	1	1	5
	Hodge (1998)	Omega-3 fatty acid rich diet and omega-3 fatty acid supplementation	1	0	1	1	1	4
	Mickleborough (2013)	Lipid extract of the New Zealand green-lipped mussel (marine lipid fraction PCSO-524™)	1	1	1	1	1	5
	Moreira (2007)	N-3 PUFA	1	1	1	1	1	5
	Schubert (2009)	N-3 PUFA-enriched fat blend	1	1	1	1	0	4
Whole food approaches	Bseikri (2018)	Nutrient dense bar (CHORI-bar)	1	0	0	0	1	2
	Papamichael (2019)	Two meals with fatty fish per week as part of the Greek Mediterranean diet	1	1	0	0	1	3
	Sexton (2013)	Mediterrenean diet	1	0	0	0	1	2
	Sudini (2016)	Broccoli sprouts	1	0	1	1	1	4
	Wood (2012)	High anti-oxidant diet	1	1	0	0	1	3

Q1 = Was the study described as randomized?; Q2 = Was the method used to generate the sequence of randomization described and appropriate?; Q3 = Was the study described as double blind?; Q4 = Was the method of double blinding described and appropriate?; Q5 = Was there a description of withdrawals and dropouts?; Points were deducted in the following cases: if the method used to generate the sequence of randomization was described and it was inappropriate or if the study was described as double blind but the method of blinding was inappropriate. Abbreviations: (LC)PUFA = (long-chain) polyunsaturated fatty acid.

Table S2. Habitual intakes of adults living in Europe and reference intakes for the interventions described in this review.

Cluster	First author (year)	Intervention	Intake habitual diet (adults)	Reference value
Herbs, herbal mixtures and extracts	Barlianto (2017)<br>Barlianto (2018)	*Nigella Sativa*	10.8 mg/day [𝕃,#]	N/A
	Salem (2017)			
	Koshak (2017)			
	Khayyal (2003)	Aqueous extract of propolis	N/A	N/A
	Hosseini (2018)<br>Zilaee (2019)	Saffron	2.3 mg/day [𝕃,##]	N/A
	Yugandhar (2017)	Extract of *B. serrata* gum resin and *A. marmelos* fruit	N/A	N/A
Supplements	Ghaffari (2014)	Vitamin E	Males: 8.2 – 16.0 mg/day<br>Females: 7.8 – 12.5 mg/day*	AI adult men and boys 10-18 y/o: 13 mg/day<br>AI adult women and girls 10-18 y/o: 11 mg/day<br>AI children 1-3 y/o: 6 mg/day<br>AI children 3-10 y/o: 9 mg/day *
	Pearson (2004)			
	Smith (2015)	Soy isoflavone	N/A	N/A
	Wood (2008)	Tomato extract and tomato juice	Tomato extract: N/A<br>Tomato juice: 963.4 mg/day [𝕃,###]	N/A
Weight loss	Dias-Junior (2014)	Low calorie intake, use of sibutramine and use of orlistat	N/A	N/A
	Jensen (2013)	Energy reduction and counselling sessions		
	Toennesen (2018)	High protein + low glycemic index diet and combination of diet and exercise		
Vitamin D3	Bar Yoseph (2015)	Vitamin D3	From diet: 1.1 – 8.2 µg/day<br>From diet and supplementation: 3.1 – 23.5 µg/day**	AI adults: 15 µg/day<br>AI children (1-17 y/o): 15 µg/day **
	Castro (2014)	Vitamin D3		
	de Groot (2015)	Vitamin D3 (Cholecalciferol)		
	Kerley (2016)	Vitamin D3		

	Martineau (2015)	Vitamin D3 (Vigantol oil)		
Omega-3 LCPUFA	Emelyanov (2002)	Lipid extract of the New Zealand green-lipped mussel	EPA and DHA from diet: 127 - 295 mg/day<br>EPA, DHA and DPA from diet and supplementation: 400 - 2570 mg/day***	AMDR: 250 – 2000 mg/day****
	Hodge (1998)	Omega-3 fatty acid rich diet and omega-3 fatty acid supplementation		
	Mickleborough (2013)	Lipid extract of the New Zealand green-lipped mussel (marine lipid fraction PCSO-524™)		
	Moreira (2007)	N-3 PUFA		
	Schubert (2009)	N-3 PUFA-enriched fat blend		
Whole food approaches	Bseikri (2018)	Nutrient dense bar (CHORI-bar)	N/A	N/A
	Papamichael (2019)	Two meals with fatty fish per week as part of the Greek Mediterranean diet		
	Sexton (2013)	Mediterrenean diet		
	Sudini (2016)	Broccoli sprouts		
	Wood (2012)	High anti-oxidant diet		

Abbreviations: AI = Adequate intake; AMDR = acceptable macronutrient distribution range; N/A = data not available; (LC)PUFA = (long-chain) polyunsaturated fatty acid; *Derived from EFSA (2015): Scientific Opinion on Dietary Reference Values for vitamin E as α-tocopherol; **Derived from EFSA (2016): Dietary reference values for vitamin D; ***Derived from EFSA (2012): Scientific opinion on the Tolerable Upper Intake Level of eicosapentaenoic acid (EPA), docosahexaenoic acid (DHA) and docosapentaenoic acid (DPA); ****Derived from FAO/WHO (2010): Expert Consultation on Fats and Fatty Acids in Human Nutrition: Fats and Fatty Acids in Human Nutrition: Report of an expert consultation; ¶ Data obtained from the EFSA Comprehensive European Food Consumption Database; #Weighed average was calculated based on available data for Austria, Belgium, Croatia, France, Ireland, Portugal, Slovenia, United Kingdom; ##Weighed average was calculated based on available data for Austria, France, Italy, Portugal, Slovenia, Spain, United Kingdom; ###Weighed average was calculated based on available data for Austria, Belgium, Croatia, Cyprus, Denmark, Estonia, Finland, France, Germany, Hungary, Ireland, Italy, Latvia, the Netherlands, Portugal, Romania, Slovenia, Spain, Sweden, United Kingdom.

Table S3. Within-group changes in lung function parameters.

First author (year)	Study group	FEV1 (%predicted)			FVC (%predicted)			PEF (%predicted)		
		BL	FU	Change	BL	FU	Change	BL	FU	Change
Herbs, herbal mixtures and extracts										
Hosseini (2018)<br>Zilaee (2019)	Saffron	76.0 ± 10.9	80.2 ± 12.6	3.0 [0.0 - 5.3] ↑	82.0 ± 12.9	84.3 ± 13.0	0.0 [-1.0 - 2.0] =			
	Placebo	76.4 ± 10.5	73.2 ± 12.2	-2.0 [-4.3 - 2.0] ↓	81.1 ± 11.7	79.7 ± 12.7	-1.0 [-4.0 - 0.3] ↓			
Khayyal (2003)	Aqueous extract of propolis	55.6 ± 2.3 [2,3]	71.6 ± 2.9 [2,3]	16.0 ↑ [2,3]	67.5 ± 1.7 [2,3]	80.5 ± 1,7 [2,3]	13.0 ↑ [2,3]	53.9 ± 2.3 [2,3]	70.4 ± 2.3 [2,3]	16.6 ↑ [2,3]
	Placebo	55.2 ± 2.4 [2,3]	55.8 ± 3.6 [2,3]	0.6 = [2,3]	73.6 ± 2.4 [2,3]	75.3 ± 2.4 [2,3]	1.7 = [2,3]	54.0 ± 1.8 [2,3]	57.5 ± 2.4 [2,3]	3.5 = [2,3]
Koshak (2017)	Nigella Sativa oil	N/A	N/A	4.0 [-1.3 - 8.8]				N/A	N/A	6.5 [0.3 - 22.8]
	Placebo	N/A	N/A	1.0 [-2.0 - 5.0]				N/A	N/A	2.0 [0.0 - 14.5]
Salem (2017)	Nigella Sativa (low dose)	85.5 ± 17.3	87.7 ± 15.8	2.2 = [1]	92.8 ± 17.3	94.8 ± 14.8	2.0 = [1]	73.5 ± 10.7 [4]	83.6 ± 8.7 [4]	10.1 ↑ [1,4]
	Nigella Sativa (high dose)	78.1 ± 21.4	85.5 ± 22.9	7.4 ↑ [1]	88.7 ± 21.9	93.0 ± 22.9	4.3 = [1]	73.7 ± 11.2 [4]	81.4 ± 8.7 [4]	7.7 ↑ [1,4]
	Placebo	81.1 ± 19.1	80.8 ± 20.6	-0.3 = [1]	90.1 ± 13.7	89.1 ± 13.7	-1.0 = [1]	76.6 ± 7.3 [4]	78.5 ± 8.8 [4]	1.9 = [1,4]

Yugandhar (2017)	Extract *of B. serrata* gum resin and *A. marmelos* fruit	1.7 ± 0.1 [3,5]	N/A	0.1 ± 0.1 [3,5]				179.4 ± 16.6 [3,5]	N/A	90.0 ± 13.0 [3,5]
	Placebo	1.6 ± 0.2 [3,5]	N/A	-0.0 ± 0.1 [3,5]				158.1 ± 16.0 [3,5]	N/A	43.9 ± 12.7 [3,5]
Supplements										
Ghaffari (2014)	Vitamine E	71.6 ± 4.1	83.1 ± 5.4	11.5 ↑ [1]	95.3 ± 2.4	100.2 ± 1.1	4.9 = [1]			
	Placebo	72.4 ± 3.4	74.8 ± 2.7	2.4 = [1]	96.1 ± 1.7	95.8 ± 1.6	-0.3 = [1]			
Pearson (2004)	Vitamin E	N/A	N/A	0.0 ± 0.2 = [5,6]	N/A	N/A	0.0 ± 0.2 = [5,6]	N/A	N/A	1.0 ± 24.0 = [5]
	Placebo	N/A	N/A	0.0 ± 0.2 = [5,6]	N/A	N/A	0.0 ± 0.3 = [5,6]	N/A	N/A	-4.0 ± 21.0 = [5]
Smith (2015)	Soy isoflavone	N/A	N/A	0.0 (-0.1; 0.1) = [5]	N/A	N/A	0.0 (-0.1; 0.0) = [5]	N/A	N/A	9.6 (-0.4; 19.6) = [5]
	Placebo	N/A	N/A	0.0 (0.0; 0.1) = [5]	N/A	N/A	0.0 (0.0; -0.1) ↑ [5]	N/A	N/A	15.8 (4.4; 27.2) = [5]
Wood (2008)	Tomato extract	76.5 (68.9; 84.1)	79.7 (72.0; 87.5) [7]	N/A	90.4 (84.3; 96.5)	91.3 (83.9; 98.7) [7]	N/A			
	Tomato juice	76.5 (68.9; 84.1)	80.0 (71.1; 88.9) [7]	N/A	90.4 (84.3; 96.5)	91.7 (83.6; 99.8) [7]	N/A			
	Placebo	76.5 (68.9; 84.1)	80.9 (72.7; 89.2) [7]	N/A	90.4 (84.3; 96.5)	92.3 (85.2; 99.4) [7]	N/A			
Weight loss										

Dias-Junior (2014)	Low calorie intake, use of sibutramine and use of orlistat	64.1 ± 3.4 [3]	70.0 ± 4.9 [3]	5.9 = [1]	82.4 ± 3.2[3]	87.8 ± 3.0 [3]	5.3 = [1]
	Placebo	59.2 ± 4.2 [3]	61.5 ± 3.2 [3]	2.3 = [1]	74.5 ± 1.5[3]	74.4 ± 2.0 [3]	-0.1 = [1]
Jensen (2013)	Energy reduction and counselling sessions	2.4 [2.0 - 2.9] [5]	N/A	0.0 [-0.2 - 0.1] = [5]	3.4 [2.7 - 3.5] [5]	N/A	0.1 ± 0.2 = [5]
	Placebo	2.6 [2.2 - 2.9] [5]	N/A	0.0 [-0.2 - 0.1] = [5]	3.3 [2.9 - 3.5] [5]	N/A	0.0 ± 0.2 = [5]
Toennesen (2018)	High protein and low glycemic index diet	87.6 ± 14.5	89.4 ± 13.4	1.8 = [1]	95.8 ± 11.5	99.2 ± 11.6	3.4 = [1]
	Combination of diet and exercise	82.6 ± 15.2	84.5 ± 16.2	1.9 = [1]	94.3 ± 15.3	96.8 ± 14.0	2.5 ↑ [1]
	Placebo	81.9 ± 12.3	81.6 ± 12.8	-0.3 = [1]	96.0 ± 12.5	95.0 ± 13.5	-1.0 = [1]
Vitamin D3							
Castro (2014)	Vitamin D3	80.7 [2]	79.7 [2]	-1.0 [1]			
	Placebo	80.4 [2]	80.1 [2]	-0.3 [1]			
de Groot (2015)	Vitamin D3 (Cholecalciferol)	99.1 ± 15.7	97.4 ± 15.7	-1.7 = [1]			
	Placebo	97.6 ± 18.1	94.0 ± 17.1	-3.6 ↓ [1]			

Kerley (2016)	Vitamin D3	105.0 [92.0 - 114.0]	N/A	-4.0 [-6.3 - (-1.0)]	94.5 [87.0 - 191.0]	N/A	-2.5 [-8.3 - 3.0]			
	Placebo	96.0 [90.0 - 104.0]	N/A	2.5 [-4.3 - 6.5]	93.0 [85.0 - 98.0]	N/A	0.0 [-5.0 - 4.5]			
Martineau (2015)	Vitamin D3 (Vigantol oil)	82.0 ± 18.7	81.6 ± 18.5	-0.4 [1]				383.0 ± 106.0 [5]	388.1 ± 116.8 [5]	5.1 [1,5]
	Placebo	81.0 ± 20.4	80.1 ± 22.8	-0.9 [1]				379.0 ± 123.0 [5]	387.7 ± 122.9 [5]	8.7 [1,5]
Omega-3 LCPUFA										
Emelyanov (2002)[8]	Lipid extract of the New Zealand green-lipped mussel	82.9 ± 4.2 [3]	82.9 ± 3.6 [3]	-0.0 ± 2.9 [3]				361.3 ± 17.4 [3,8]	408.3 ± 18.7 [3,8]	47.0 ± 11.7 [3,8]
	Placebo	92.3 ± 2.9 [3]	90.5 ± 3.2 [3]	-1.8 ± 4.4 [3]				384.3 ± 21.5 [3,8]	350.9 ± 21.3 [3,8]	-33.4 ± 6.2 [3,8]
Emelyanov (2002)[9]	Lipid extract of the New Zealand green-lipped mussel	82.9 ± 4.2 [3]	82.9 ± 3.6 [3]	-0.0 ± 2.9 [3]				375.4 ± 18.2 [3,9]	406.5 ± 19.7 [3,9]	31.1 ± 14.6 [3,9]
	Placebo	92.3 ± 2.9 [3]	90.5 ± 3.2 [3]	-1.8 ± 4.4 [3]				399.6 ± 16.7 [3,9]	403.9 ± 18.3 [3,9]	4.3 ± 10.3 [3,9]
Hodge (1998)	Omega-3 fatty acid rich diet and omega-3 fatty acid supplementation	81.1 (75.3; 86.9)	83.7 (78.4; 89.0)	2.6 [1]						

	Placebo	86.1 (79.1; 93.1)	83.5 (78.3; 88.7)	-2.6 [1]						
Mickleborough (2013)	Marine lipid fraction PCSO-524™							N/A	386.3 ± 22.8 [5]	N/A
	Placebo							N/A	364.5 ± 17.2 [5]	N/A
Moreira (2007)	N-3 PUFA	96.7 (85.4; 108.0)	100.7 (87.9; 113.6)	4.0 (-3.7; 11.7) =						
	Placebo	90.9 (75.9; 105.8)	94.5 (75.9; 113.0)	3.7 (-4.6; 12.9) =						
Schubert (2009)	N-3 PUFA-enriched fat blend	4.3 ± 0.3 [2,3,5]	4.3 ± 0.3 [2,3,5]	0.0 [1,5]						
	Placebo	4.1 ± 0.2 [2,3,5]	4.2 ± 0.2 [2,3,5]	0.1 [1,5]						
Whole food approaches										
Bseikri (2018)	Nutrient dense bar (CHORI-bar)	92.3 ± 15.6	97.5 ± 13.2	5.2 = [1]	110.5 ± 14.0	115.9 ± 12.1	5.4 = [1]			
	Placebo	97.4 ± 16.1	96.9 ± 16.3	-0.6 = [1]	113.0 ± 17.4	114.5 ± 18.7	1.5 = [1]			
Papamichael (2019)	Two meals with fatty fish per week as part of the Greek MD	97.2 ± 8.8	100.2 ± 9.4	2.8	94.6 ± 8.7	96.9 ± 9.2	2.5	94.3 ± 19.3	100.6 ± 21.0	6.1
	Placebo	99.1 ± 10.6	100.1 ± 8.8	0.6	96.3 ± 11.1	96.8 ± 9.1	-0.1	93.5 ± 18.8	101.2 ± 21.7	7.1

Sexton (2013)	High-intervention MD	N/A	N/A	0.1 ± 0.1 [3,5]	N/A	N/A	0.1 ± 0.1 [3,5]
	Low intervention MD	N/A	N/A	0.0 ± 0.1 [3,5]	N/A	N/A	0.0 ± 0.1 [3,5]
	Placebo	N/A	N/A	0.0 ± 0.1 [3,5]	N/A	N/A	0.0 ± 0.1 [3,5]
Sudini (2016)	Broccoli sprouts	3.0 ± 0.8	3.0 ± 0.8	0.0 ± 0.1	4.0 ± 0.9	3.90 ± 0.86	-0.05 ± 0.10
	Placebo	2.9 ± 0.9	2.9 ± 0.9	-0.0 ± 0.2	3.8 ± 1.0	3.8 ± 1.0	0.0 ± 0.2

Values are presented as mean ± SD, mean (lower bound 95% CI; upper bound 95% CI), median [Q1 – Q3] or median [minimum; maximum]. = indicates that within-group changes were not significantly different from baseline; ↑ indicates a significant increase compared to baseline; ↓ indicates a significant decrease compared to baseline; blank indicates that within-group changes were not reported in the article; N/A indicates data could not be extracted. Abbreviations: BL = baseline; FU = follow-up; FEV_1 = forced expiratory flow in one second; FVC = forced vital capacity; PEF = peak expiratory flow; (LC)PUFA = (long-chain) polyunsaturated fatty acid; MD = Mediterranean diet; 1 = calculated; 2 = estimated using pixel ruler; 3 = ± SEM; 4 = PEF variability; 5 = unit is liters; 6 = unit transformed; 7 = n at follow-up is smaller than n at baseline; 8 = morning PEF; 9 = evening PEF.

Table S4. Within-group changes in asthma control and quality of life.

First author (year)	Study group	Asthma control						Quality of life		
		ACT			ACQ			(P)AQLQ		
		BL	FU	Change	BL	FU	Change	BL	FU	Change
Herbs, herbal mixtures and extracts										
Barlianto (2017)*, Barlianto (2018)*	Nigella Sativa oil	16.6 ± 2.53	20.3 ± 1.82	3.7 ↑ [1]						
	Placebo	17.6 ± 1.22	19.4 ± 1.15	1.8 ↑ [1]						
Koshak (2017)	Nigella Sativa oil	16.0 ± 3.9	21.1 ± 2.6	5.1 [1]						
	Placebo	16.6 ± 3.6	19.6 ± 3.7	3.0 [1]						
Salem (2017)	Nigella Sativa (low dose)	17.5 ± 1.3 [2]	21.1 ± 2.1 [2]	3.6 ↑ [1]						
	Nigella Sativa (high dose)	17.4 ± 1.4 [2]	21.1 ± 1.6 [2]	3.6 ↑ [1]						
	Placebo	17.2 ± 1.3 [2]	19.4 ± 2.2 [2]	2.2 ↑ [1]						
Yugandhar (2017)	Extract of *B. serrata* gum resin and *A. marmelos* fruit							25.4 [4]	N/A	16.2 [4]
	Placebo							25.1 [4]	N/A	5.4 [4]
Supplements										
Smith (2015)	Soy isoflavone	N/A	N/A	2.2 (1.5; 2.9) =						
	Placebo	N/A	N/A	2.0 (1.4; 2.5) =						
Wood (2008)	Tomato extract				1.4 (1.0; 1.8)	1.1 (0.8; 1.5) [5]	N/A			
	Tomato juice				1.4 (1.0; 1.8)	1.0 (0.6; 1.31) [5]	N/A			
	Placebo				1.4 (1.0; 1.8)	1.1 (0.8; 1.4) [5]	N/A			

Weight loss										
Dias-Junior (2014)	Low calorie intake, use of sibutramine and use of orlistat	12.3 ± 1.1 [3]	17.4 ± 1.1 [3]	5.2 ↑ [1]	3.0 ± 0.3 [3]	1.6 ± 0.2 [3]	-1.4 ↓ [1]			
	Placebo	11.2 ± 1.2 [3]	12.1 ± 0.7 [3]	0.9 = [1]	2.9 ± 0.3 [3]	2.9 ± 0.2 [3]	-0.0 = [1]			
Jensen (2013)	Energy reduction and counselling sessions				N/A	N/A	-0.4 ± 0.5 ↓	5.5 [4.7 - 6.3]	N/A	0.7 ± 1.2 =
	Placebo				N/A	N/A	0.1 ± 0.5 ↑	6.0 [5.7 - 6.5]	N/A	0.1 ± 0.7 =
Toennesen (2018)	High protein and low glycemic index diet				2.0 ± 0.6	1.3 ± 0.8	-0.7 ↓ [1]	5.3 ± 0.8	5.9 ± 0.9	0.6 ↑ [1]
	Combination of diet and exercise				1.9 ± 0.7	1.0 ± 0.7	-0.9 ↓ [1]	5.2 ± 0.8	6.2 ± 0.7	1.0 ↑ [1]
	Placebo				1.8 ± 0.8	1.5 ± 0.8	-0.3 ↓ [1]	5.2 ± 0.8	5.7 ± 0.7	0.5 ↑ [1]
Vitamin D3										
Castro (2014)	Vitamin D3	N/A	N/A	0.5 (-0.1; 1.2)						
	Placebo	N/A	N/A	-0.1 (-0.1; 0.0)						
de Groot (2015)	Vitamin D3 (Cholecalciferol)				0.9 [0.4 - 1.9]	0.8 [0.4 - 1.3]	-0.1 = [1]	6.0 [5.1 - 6.4]	6.3 [6.0 - 6.6]	0.3 ↑ [1]
	Placebo				1.2 [0.7 - 1.6]	1.1 [0.8 - 1.6]	-0.1 = [1]	5.7 [5.2 - 6.3]	6.0 [5.6 - 6.2]	0.3 = [1]
Kerley (2016)	Vitamin D3	19.0 [17.0 - 21.0]	N/A	2.0 [-2.0 - 4.0]				5.6 [5.0 - 6.2]	N/A	0.5 [-0.2 - 0.8]
	Placebo	17.0 [14.3 - 19.0]	N/A	3.5 [0.0 - 5.0]				5.4 [3.8 - 6.0]	N/A	0.9 [-0.3 - 1.5]
Martineau (2015)	Vitamin D3 (Vigantol oil)	19.2 ± 3.9	20.4 ± 4.0	1.2 [1]						
	Placebo	18.9 ± 3.9	20.4 ± 4.2	1.5 [1]						
Omega-3 LCPUFA										

Moreira (2007)	N-3 PUFA				1.4 (0.8; 2.1)	1.0 (0.4; 1.5)	-0.5 (-0.9; -0.1) ↓			
	Placebo				1.7 (1.0; 2.5)	1.1 (0.4; 1.8)	-0.6 (-1.2; -0.1) ↓			
Whole food approaches										
Bseikri (2018)	Nutrient dense bar (CHORI-bar)	15.0 ± 3.0	20.3 ± 3.1	5.3 ↑ [1]						
	Placebo	13.4 ± 3.4	19.7 ± 3.2	6.3 ↑ [1]						
Papamichael (2019)	Two meals with fatty fish per week as part of the Greek MD				0.4 ± 0.3	0.2 ± 0.5	-0.1	6.8 ± 0.3	6.8 ± 0.6	0.1
	Placebo				0.4 ± 0.4	0.2 ± 0.3	-0.2	6.7 ± 0.4	6.9 ± 0.2	0.2
Sexton (2013)	High-intervention MD	N/A	N/A	-0.2 ± 0.2 [3]				N/A	N/A	0.5 ± 0.2 [3]
	Low intervention: MD	N/A	N/A	-0.1 ± 0.2 [3]				N/A	N/A	0.2 ± 0.2 [3]
	Placebo	N/A	N/A	-0.1 ± 0.2 [3]				N/A	N/A	0.2 ± 0.2 [3]
Sudini (2016)	Broccoli sprouts	21.0 [20.0 - 22.0]	21.0 [19.0 - 22.0]	0.0 [-1.0 - 1.3]						
	Placebo	20.0 [18.0 - 23.0]	22.0 [20.0 - 23.0]	0.0 [0.0 - 1.5]						
Wood (2012)	High anti-oxidant diet				0.7 [0.4 - 1.4]	0.9 [0.4 - 1.4]	0.2 = [1]			
	Placebo				0.9 [0.4 - 1.4]	0.9 [0.4 - 1.6]	0.0 = [1]			

Values are presented as mean ± SD, mean (lower bound 95% CI; upper bound 95% CI), median [Q1 – Q3]. = indicates that within-group changes were not significantly different from baseline; ↑ indicates a significant increase compared to baseline; ↓ indicates a significant decrease compared to baseline; blank indicates that within-group changes were not reported in the article; N/A indicates data could not be extracted. Abbreviations: BL = baseline; FU = follow-up; ACT = asthma control test; ACQ = asthma control questionnaire; (P)AQLQ = (pediatric) asthma quality of life questionnaire; (LC)PUFA = (long-chain) polyunsaturated fatty acid; MD = Mediterranean diet; 1 = calculated; 2 = estimated using pixel ruler; 3 = ± SEM; 4 = total score calculated from domain scores; 5 = n at follow-up is smaller than n at baseline.

Table S5. Within-group changes in immunological parameters in breath and immune cells.

**First author (year)**	**Study group**	**Breath**						**Cells**					
		**FeNO (ppb)**			**sEOS (%)**			**sNEU (%)**			**bEOS (10⁹ cells/L)**		
		BL	FU	Change	BL	FU	Change	BL	FU	Change	BL	FU	Change
Herbs, herbal mixtures and extracts													
Koshak (2017)	Nigella Sativa oil										N/A	N/A	-0.1 [-0.2 – 0.0] [1]
	Placebo										N/A	N/A	0.0 [-0.1 - 0.1] [1]
Salem (2017)	Nigella Sativa (low dose)	23.0 ± 13.3	18.1 ± 8.2	-4.9 ↓ [2]									
	Nigella Sativa (high dose)	27.6 ± 30.60	26.9 ± 29.1	-0.7 = [2]									
	Placebo	34.9 ± 32.8	34.8 ± 26.9	-0.1 = [2]									
Hosseini (2018) Zilaee (2019)	Saffron										5.6 [3.0 - 7.5] [3]	4.55 [2.8 - 6.0] [3]	-0.85 [-1.62 - 0.15] ↓ [3]
	Placebo										4.9 [3.9 - 6.5] [3]	4.9 [3.0 - 6.0] [3]	0.1 [-1.6 - 0.6] = [3]
Supplements													
Smith (2015)	Soy isoflavone	N/A	N/A	1.4 (-1.7; 4.5) =							N/A	N/A	0.0 (0.0; 0.0) = [1]
	Placebo	N/A	N/A	-3.5 (-6.0; -1.0) ↓							N/A	N/A	0.0 (0.0; 0.0) = [1]
Wood (2008)	Tomato extract	19.9 (16.4; 27.5)	19.6 (13.1; 31.6) [4]	N/A	1.0 (0.0; 3.1)	0.9 (0.1; 1.8) [4]	N/A	41.0 (24.2; 56.6)	39.8 (18.4; 77.5) [4]	N/A			
	Tomato juice	19.9 (16.4; 27.5)	19.7 (11.0; 25.9) [4]	N/A	1.0 (0.0; 3.1)	0.9 (0.0; 17.8) [4]	N/A	41.0 (24.2; 56.6)	42.0 (21.0; 67.8) [4]	N/A			

	Placebo	19.9 (16.4; 27.5)	19.1 (12.9; 31.4) [4]	N/A	1.0 (0.0; 3.1)	0.4 (0.0; 1.5) [4]	N/A	41.0 (24.2; 56.6)	55.1 (35.0; 91.1) [4]	N/A			
Weight loss													
Dias-Junior (2014)	Low calorie intake, use of sibutramine and use of orlistat	19.6 ± 3.7	26.8 ± 5.2	7.2 = [2]	15.0 ± 4.9	14.4 ± 4.7	-0.6 = [2]	40.2 ± 5.8	41.9 ± 7.3	1.7 = [2]			
	Placebo	20.1 ± 4.9	19.3 ± 3.2	-0.8 = [2]	11.9 ± 3.9	12.5 ± 3.2	0.6 = [2]	41.7 ± 4.0	53.6 ± 4.1	11.9 = [2]			
Jensen (2013)	Energy reduction and counselling sessions	13.1 [8.4 - 41.8]	N/A	-2.6 [-11.3 - 0.4] =	0.8 [0.5 - 5.3]	N/A	-0.1 [-0.5 - 5.1] =	10.5 [8.0 - 18.8]	N/A	-4.8 [-7.5 – (-0.6)] =			
	Placebo	27.2 [10.5 - 46.7]	N/A	-1.9 [-4.0 - 0.3] =	0.8 [0.3 - 8.5]	N/A	0.0 [-0.8 - 2.3] =	10.3 [2.8 - 27.5]	N/A	1.0 [-4.5 - 14.0] =			
Toennesen (2018)	High protein and low glycemic index diet	20.5 (13.0)	18.0 (19.5)	-2.5 = [2]	0.5 (5.8)	5.5 (2.5)	0.0 = [2]	54.3 (26.0)	61.0 (45.5)	6.7 = [2]	0.2 (0.2)	0.1 (0.2)	0.0 = [2]
	Combination of diet and exercise	32.5 (29.0)	27.0 (32.3)	-5.5 = [2]	7.8 (14.9)	4.80 (13.1)	-3.0 = [2]	43.0 (52.4)	46.3 (33.6)	3.3 = [2]	0.21(0.1)	0.2 (0.1)	0.0 = [2]
	Placebo	20.8 (35.6)	20.3 (22.3)	-0.5 = [2]	1.5 (6.7)	0.8 (5.2)	-0.7 = [2]	60.3 (41.0)	55.4 (33.5)	-4.9 = [2]	0.2 (0.2)	0.2 (0.3)	0.0 = [2]
Vitamin D3													
Bar Yoseph (2015)	Vitamin D	36.6 ± 39.1	34.2 ± 26.8	-2.4 = [2]							0.6 ± 0.9	0.3 ± 0.2	-0.2 = [1,2]
	Placebo	58.6 ± 54.7	51.0 ± 40.2	-7.6 = [2]							0.4 ± 0.3	0.3 ± 0.3	-0.1 = [1,2]
Castro (2014)	Vitamin D3				N/A	N/A	-0.3 (-1.4; 0.8)						
	Placebo				N/A	N/A	0.2 (-0.9; 1.3)						
de Groot (2015)	Vitamin D3 (Cholecalciferol)	24.0 [19.0 -36.0]	22.0 [17.0 - 29.0]	-2.0 = [2]	3.1 [0.3 - 13.3]	0.7 [0.2 - 11.4]	-2.4 = [2]	64.8 [44.6 - 76.4]	65.5 [45.1 - 86.2]	0.7 = [2]	0.2 [0.1 - 0.3]	0.2 [0.1 - 0.3]	0.0 = [2]
	Placebo	33.0 [15.0 -67.0]	26.0 [11.0 - 60.0]	-7.0 = [2]	6.7 [0.2 - 39.7]	3.9 [0.2 - 50.9]	-2.8 = [2]	67.8 [33.8 - 75.6]	50.5 [22.5 - 72.1]	-17.3 = [2]	0.2 [0.1 - 0.4]	0.2 [0.1 - 0.4]	0.0 = [2]
Martineau (2015)	Vitamin D3 (Vigantol oil)	38.1 ± 29.1	37.5 ± 26.9	-0.6 [2]									
	Placebo	37.0 ± 26.0	38.5 ± 36.9	1.5 [2]									

Omega-3 LCPUFA													
Hodge (1998)	Omega-3 fatty acid rich diet and omega-3 fatty acid supplementation										0.9 [0.5 - 1.2]	0.7	-0.3 [-0.3 – 0.0] [2,5]
	Placebo										0.6 [0.6 - 0.9]	0.8	0.2 [-0.1 - 0.3] [2,5]
Mickleborough (2013)	Marine lipid fraction PCSO-524™	N/A	15.3 ± 10.7	N/A									
	Placebo	N/A	25.2 ± 19.1	N/A									
Moreira (2007)	N-3 PUFA	27.6 (16.6; 38.6)	30.0 (15.8; 44.2)	2.4 (-3.5; -8.3) =									
	Placebo	20.4 (10.0; 30.1)	25.0 (12.3; 37.7)	4.6 (0.2; 8.9)↑									
Schubert (2009)	N-3 PUFA-enriched fat blend	N/A	N/A	-2.1 ± 3.6	1.5 [5]	0.3 [5]	-1.2 [2]				4.4 ± 0.6 [3,5]	3.8 ± 0.5 [3,5]	-0.6 [2,3]
	Placebo	N/A	N/A	10.8 ± 3.1	1.5 [5]	0.7 [5]	-0.8 [2]				6.0 ± 0.7 [3,5]	5.6 ± 0.9 [3,5]	-0.5 [2,3]
Whole food approaches													
Bseikri (2018)	Nutrient dense bar (CHORI-bar)	35.8 ± 23.5	30.5 ± 20.2	-5.3 = [2]									
	Placebo	24.0 ± 11.5	22.0 ± 13.9	-2.0 = [2]									
Papamichael (2019)	Two meals with fatty fish per week as part of the Greek MD	17.9 ± 17.6	14.6 ± 15.1	-3.8									
	Placebo	10.2 ± 7.2	18.1 ± 29.4	8.1									
Sexton (2013)	High-intervention MD										N/A	N/A	-0.0 ± 0.0 [6]
	Low intervention MD										N/A	N/A	0.0 ± 0.0 [6]

	Placebo										N/A	N/A	-0.1 ± 0.1 [6]
Sudini (2016)	Broccoli sprouts	21.0 [15.0 - 42.0]	22.0 [15.9 - 34.5] [2]	1.0 [2]									
	Placebo	25.5 [15.0 - 42.0]	19.50 [17.0 - 45.3] [2]	-6.0 [2]									
Wood (2012)	High anti-oxidant diet	17.0 [12.0 -30.0]	19.0 [15.0 - 31]	2.0 = [2]	1.0 [0.3 - 2.8]	0.8 [0.3 - 2.3]	-0.2 = [2]	46.1 ± 26.2	42.0 ± 26.8	-4.1 = [2]			
	Placebo	23.0 [15.0 -38.0]	24.0 [16.0 - 35.0]	1.0 = [2]	1.8 [0.5 - 6.4]	1.3 [0.3 - 5.5]	-0.5 ↓ [2]	42.0 ± 20.9	45.7 ± 19.9	3.7 = [2]			

Values are presented as mean ± SD, mean (lower bound 95% CI; upper bound 95% CI), median [Q1 – Q3] or median (IQR). = indicates that within-group changes were not significantly different from baseline; ↑ indicates a significant increase compared to baseline; ↓ indicates a significant decrease compared to baseline; blank indicates that within-group changes were not reported in the article; N/A indicates data could not be extracted. Abbreviations: BL = baseline; FU = follow-up; FeNO = fractional exhaled nitric oxide; sEOS = sputum eosinophils; sNEU = sputum neutrophils; bEOS = blood eosinophils; PUFA = (long-chain) polyunsaturated fatty acid; MD = Mediterranean diet; 1 = unit transformed; 2 = calculated; 3 = unit is percentage; 4 = n at follow-up is smaller than n at baseline; 5 = estimated using pixel ruler; 6 = ± SEM.

Table S6. Within-group changes in Th1 and Th2 cytokines and IgE.

First author (year)	Study group	Th1 cytokines			Th2 cytokines and IgE					
		IFN-γ (pg/ml)			IL-4 (pg/ml)			IgE (IU/ml)		
		BL	FU	Change	BL	FU	Change	BL	FU	Change
Herbs, herbal mixtures and extracts										
Barlianto (2017) Barlianto (2018)	Nigella Sativa oil	12.5 ± 4.4	20.0 ± 6.4	7.5	1.4 ± 0.3	1.1 ± 0.2	-0.3 [1]			
	Placebo	10.1 ± 2.2	9.8 ± 3.3	-0.3	1.3 ± 0.5	1.4 ± 0.5	0.1 [1]			
Koshak (2017)	Nigella Sativa oil							N/A	N/A	-0.7 [-25.2 - 6.3] [3]
	Placebo							N/A	N/A	-10.0 [-50.6 - 5.7] [3]
Salem (2017)	Nigella Sativa (low dose)	3.8 ± 5.8	4.7 ± 6.0	0.9 ↑ [1]	2.4 ± 6.7	2.3 ± 6.8	-0.1 = [1]	4.5x10^5 ± 7.1x10^5 [5]	3.9x10^5 ± 6.2x10^5 [5]	-6.0 x10^4 = [1,5]
	Nigella Sativa (high dose)	2.8 ± 5.8	3.3 ± 6.0	0.5 ↑ [1]	2.2 ± 6.5	2.1 ± 6.4	-0.1 = [1]	3.9x10^5 ± 4.7x10^5 [5]	3.2x10^5 ± 3.7x10^5 [5]	-7.2 x10^4 ↓ [1,5]
	Placebo	3.0 ± 5.5	2.6 ± 5.4	-0.4 = [1]	1.6 ± 5.7	1.6 ± 5.7	-0.0 = [1]	6.2 x10^5 ± 8.0x10^5 [5]	6.0x10^5 ± 7.5x10^5 [5]	-2.8 x10^4 = [1,5]
Yugandhar (2017)	Extract of *B. serrata* gum resin and *A. marmelos* fruit	12.7 ± 0.6 [6]	22.0 ± 1.4 [6]	1.8 [1,6]	1.6 ± 0.2 [6]	1.1 ± 0.2 [6]	-0.5 [1,6]			
	Placebo	13.6 ± 0.5 [6]	15.4 ± 0.4 [6]	9.4 [1,6]	1.5 ± 0.2 [6]	1.4 ± 0.2 [6]	-0.1 [1,6]			
Supplements										
Ghaffari (2014)	Vitamine E							154.5 ± 33.8	118.3 ± 14.4	-36.2 = [1]
	Placebo							147.2 ± 27.6	127.0 ± 22.3	-20.2 = [1]
Pearson (2004)	Vitamin E							N/A	N/A	1.0 ± 1.2 = [6]
	Placebo							N/A	N/A	1.0 ± 1.5 = [6]
Weight loss										

Dias-Junior (2014)	Low calorie intake, use of sibutramine and use of orlistat							24.4 ± 6.5	23.80 ± 6.3	-0.6 = [1]
	Placebo							22.4 ± 5.9	17.7 ± 5.3	-4.7 = [1]
Vitamin D3										
Bar Yoseph (2015)	Vitamin D							432.8 ± 465.7	398.1 ± 412.6	-34.7 = [1]
	Placebo							433.8 ± 455.0	539.5 ± 651.8	105.7 = [1]
de Groot (2015)	Vitamin D3 (Cholecalciferol)							29.0 [13.0 - 117.0] [5]	29.0 [13.0 - 88.0] [5]	0.0 = [1,5]
	Placebo							69.0 [1.0 - 2110.0] [5]	47.0 [4.0 - 264.0] [5]	-22.0 = [1,5]
Whole food approaches										
Bseikri (2018)	Nutrient dense bar (CHORI-bar)							518.8 ± 712.2	560.0 ± 734.2	41.5 = [1]
	Placebo							294.3 ± 516.6	247.3 ± 497.2	-47.0 ↓ [1]
Sexton (2013)	High-intervention MD	N/A	N/A	-0.8 ± 0.3 [6]						
	Low intervention MD	N/A	N/A	-0.6 ± 0.4 [6]						
	Placebo	N/A	N/A	-0.4 ± 0.4 [6]						
Sudini (2016)	Broccoli sprouts				1.4 [0.8 - 2.3]	2.2 [1.1 - 3.1]	0.6 [0.1 - 1.0]			
	Placebo				1.8 [0.7 - 2.2]	2.0 [1.3 - 2.6]	0.5 [0.3 - 1.0]			

Values are presented as mean ± SD, median [Q1 – Q3] or median [minimum; maximum]. = indicates that within-group changes were not significantly different from baseline; ↑ indicates a significant increase compared to baseline; ↓ indicates a significant decrease compared to baseline; blank indicates that within-group changes were not reported in the article; N/A indicates data could not be extracted. Abbreviations: BL = baseline; FU = follow-up; Th = T helper cell; Ig = immunoglobulin; IFN = interferon; IL = interleukin; MD = Mediterranean diet; 1 = calculated; 2 = ELISA units; 3 = estimated using pixel ruler; 4 = unit is ng/ml; 5 = unit transformed; 6 = ± SEM; 7 = unit was missing in original article and was estimated based on other articles of the same research group.

Table S7. Within-group changes in Treg cytokines and pro-inflammatory markers.

First author (year)	Study group	Treg cytokines			Pro-inflammatory markers					
		IL-10 (pg/ml)			IL-6 (pg/ml)			CRP (mg/L)		
		BL	FU	Change	BL	FU	Change	BL	FU	Change
Herbs, herbal mixtures and extracts										
Hosseini (2018) Zilaee (2019)	Saffron							0.1 [0.0 - 0.1] [1]	0.0 [0.0 - 0.1] [1]	-0.0 [-0.0 – 0.0] ↓[1]
	Placebo							0.1 [0.0 - 0.1] [1]	0.1 [0.0 - 0.1] [1]	-0.0 [-0.0 - 0.0] = [1]
Khayyal (2003)	Aqueous extract of propolis	88.1 ± 14.3 [2,3]	273.8 ± 35.7 [2,3]	185.7 ↑ [2,3,4]	59.1 ± 7.1 [2,3]	33.5 ± 3.5 [2,3]	-25.6 ↓ [2,3,4]			
	Placebo	100.0 ± 19.1 [2,3]	142.9 ± 28.6 [2,3]	42.9 ↑ [2,3,4]	88.2 ± 17.7 [2,3]	109.4 ± 29.1 [2,3]	-3.0 = [2,3,4]			
Salem (2017)	Nigella Sativa (low dose)	2.4 ± 6.1	2.8 ± 6.3	0.4 = [4]						
	Nigella Sativa (high dose)	1.7 ± 6.6	1.5 ± 6.6	-0.2 = [4]						
	Placebo	2.2 ± 5.8	1.6 ± 6.3	-0.6 = [4]						
Supplements										
Smith (2015)	Soy isoflavone				N/A	N/A	1.0 (0.9; 1.1) =	N/A	N/A	1.0 (0.9; 1.1) =
	Placebo				N/A	N/A	1.0 (0.9; 1.1) =	N/A	N/A	1.0 (0.9; 1.2) =
Weight loss										
Dias-Junior (2014)	Low calorie intake, use of sibutramine and use of orlistat							286.5 ± 62.0	292.8 ± 80.7	24.3 = [4]
	Placebo							409.1 ± 107.0	507.5 ± 124.3	98.4 = [4]
Jensen (2013)	Energy reduction and counselling sessions				1.2 [0.7 - 2.7]	N/A	0.3 [-0.3 - 0.4] =	2.1 [1.5 - 3.3]	N/A	-0.4 [-0.5 - 0.4] =
	Placebo				1.4 [0.7 - 2.0]	N/A	-0.1 [-0.5 - 0.4] =	2.1 [0.7 - 4.0]	N/A	0.7 [-0.1 - 1.9] ↑

Toennesen (2018)	High protein and low glycemic index diet				1.5 (1.0)	1.3 (0.7)	-0.1 = [4]	1.1 (1.6)	0.9 (1.8)	-0.2 = [4]
	Combination of diet and exercise				1.5 (0.8)	1.6 (0.9)	0.1 = [4]	0.9 (2.1)	1.2 (1.4)	0.3 = [4]
	Placebo				1.70 (1.6)	1.5 (0.8)	-0.2 = [4]	1.1 (2.0)	1.1 (1.3)	-0.1 = [4]
Vitamin D3										
Bar Yoseph (2015)	Vitamin D							2.0 ± 1.0	2.3 ± 1.7	0.3 = [4]
	Placebo							2.1 ± 1.3	2.1 ± 0.9	0.1 = [4]
Kerley (2016)	Vitamin D3	1.1x10^{5} [8.9x10^{4} - 1.3x10^{5}]	N/A	-1.3x10^{4} [-2.5x10^{4} - 3.0x10^{3}]				0.3 [0.2 - 0.6]	N/A	0.1 [-0.1 - 0.9]
	Placebo	1.1x10^{5} [8.6x10^{4} - 1.5x10^{5}]	N/A	-1.7x10^{4} [-2.6x10^{4} – (-2.7x10^{4})]				0.8 [0.5 - 1.5]	N/A	-0.4 [-0.9 - 0.2]
Whole food approaches										
Bseikri (2018)	Nutrient dense bar (CHORI-bar)							3.9 ± 4.3	4.4 ± 6.1	0.5 = [4]
	Placebo							3.8 ± 5.7	3.5 ± 4.9	-0.4 = [4]
Sexton (2013)	High-intervention MD	N/A	N/A	-0.1 ± 0.6 [3]	N/A	N/A	-1.2 ± 0.7 [3]	N/A	N/A	2.1 ± 1.9 [3]
	Low intervention MD	N/A	N/A	-0.4 ± 0.6 [3]	N/A	N/A	-0.5 ± 0.7 [3]	N/A	N/A	2.9 ± 1.7 [3]
	Placebo	N/A	N/A	-0.2 ± 0.6 [3]	N/A	N/A	-0.3 ± 0.7 [3]	N/A	N/A	-0.6 ± 1.7 [3]
Sudini (2016)	Broccoli sprouts				0.6 [0.1 - 1.6]	0.7 [0.0 - 1.4]	-0.1 [-0.6 - 0.0]			
	Placebo				1.6 [0.5 - 2.6]	1.3 [0.4 - 2.8]	0.0 [-1.5 - 0.5]			
Wood (2012)	High anti-oxidant diet				1.9 [1.1 - 2.2]	1.9 [1.3 - 2.5]	0.0 = [4]	4.2 [0.9 - 9.1]	3.0 [1.3 - 9.5]	-1.2 = [4]
	Placebo				1.9 [1.3 - 3.0]	2.0 [1.3 - 2.9]	0.1 = [4]	2.5 [1.1 - 6.0]	3.3 [1.5 - 6.4]	0.8 = [4]

Values are presented as mean ± SD, mean (lower bound 95% CI; upper bound 95% CI), median [Q1 – Q3], or median (IQR). = indicates that within-group changes were not significantly different from baseline; ↑ indicates a significant increase compared to baseline; ↓ indicates a significant decrease compared to baseline; blank indicates that within-group changes were not reported in the article; N/A indicates data could not be extracted. Abbreviations: BL = baseline; FU = follow-up; Treg = regulatory T-cell; IL = interleukin; CRP = C-reactive protein; MD = Mediterranean diet; 1 = unit transformed; 2 = estimated using pixel ruler; 3 = ± SEM; 4= caculated.
